# Pathogenesis of Fistulating Crohn’s Disease: A Review

**DOI:** 10.1016/j.jcmgh.2022.09.011

**Published:** 2022-09-29

**Authors:** Colleen Georgette Chantelle McGregor, Ruchi Tandon, Alison Simmons

**Affiliations:** 1 MRC Human Immunology Unit, MRC Weatherall Institute of Molecular Medicine, University of Oxford, Oxford, United Kingdom; 2 Translational Gastroenterology Unit, John Radcliffe Hospital, Headington, Oxford, United Kingdom; 3 Pathology, Department of Cellular Pathology, Oxford University Hospitals NHS Foundation Trust, Oxford, United Kingdom

**Keywords:** Crohn’s-associated Fistula, Epithelial-to-mesenchymal Transition, Model Systems, Penetrating, CD, Crohn’s disease, CLPF, colonic lamina propria fibroblast, DKK-1, Dickkopf-related protein 1, ECM, extracellular matrix, EMT, epithelial-to-mesenchymal transition, Ets, E-twenty-six, IBD, inflammatory bowel disease, IEC, intestinal epithelial cell, IEO, intestinal epithelial organoid, IL, interleukin, MMP, matrix metalloproteinase, NOD2, nucleotide oligomerization domain 2, OR, odds ratio, TC, transitional cell, TGF-β, transforming growth factor-beta, TIMP, tissue inhibitor of MMP, TNF, tumor necrosis factor, TNF-R1, TNF receptor 1

## Abstract

Sustained, transmural inflammation of the bowel wall may result in the development of a fistula in Crohn’s disease (CD). Fistula formation is a recognized complication and cause of morbidity, occurring in 40% of patients with CD. Despite advanced treatment, one-third of patients experience recurrent fistulae. Development of targeting treatment for fistulae will be dependent on a more in depth understanding of its pathogenesis. Presently, pathogenesis of CD-associated fistulae remains poorly defined, in part due to the lack of accepted in vitro tissue models recapitulating the pathogenic cellular lesions linked to fistulae and limited in vivo models. This review provides a synthesis of the existing knowledge of the histopathological, immune, cellular, genetic, and microbial contributions to the pathogenesis of CD-associated fistulae including the widely accredited contribution of epithelial-to-mesenchymal transition, upregulation of matrix metalloproteinases, and overexpression of invasive molecules, resulting in tissue remodeling and subsequent fistula formation. We conclude by exploring how we might utilize advancing technologies to verify and broaden our current understanding while exploring novel causal pathways to provide further inroads to future therapeutic targets.

**Synopsis**

Fistulating Crohn’s disease is a complex clinical entity; its pathogenesis appears multifactorial – a combination of epithelial-to-mesenchymal transition, upregulated matrix metalloproteinases, and pro-inflammatory cytokines, together with genetic and microbial contributions. Development of in vivo models are required to verify and broaden current understanding.

Fistulae formation is a recognized complication and cause of morbidity in Crohn’s disease (CD). Chronic, transmural inflammation of the bowel wall in CD, can result in the development of sinus tracts that, once penetrating the serosa, can result in a fistula (an abnormal communication between two epithelialized surfaces). They can be defined anatomically; those arising internally and those involving the perineum. Internal fistulae may be further classified into those forming an internal communication with another bowel layer (ie, enteroenteric) or a communication between the intestine and other organs (ie, enterocutaneous or enterovesical).^1^ About 35% to 40% of patients will develop at least one fistula throughout their disease course.^2^^,^^3^ The majority of fistulae are perianal (54%); however, the less frequent, internal fistula is more difficult to diagnose and treat.^2^ Ethnic differences are also observed with this phenotype^4^^,^^5^; South Asian patients with CD are more likely to develop penetrating disease when compared with Caucasians (24.1% vs 8.6%).^6^

One-third of patients will experience recurring fistulae despite advanced medical therapies and surgical interventions.^2^ Therefore, novel therapeutic approaches are required to treat fistulating CD; however, the prerequisite to this is a better understanding of its pathogenesis.

The pathogenesis of Crohn’s-associated fistulae is poorly defined, in part as accessibility to human tissue from fistulating disease is limiting. In addition, as yet, there are no accepted in vitro tissue models recapitulating the pathogenic cellular lesions linked to fistulae and limited in vivo models to study this phenomenon. Available literature describes the transition of intestinal epithelial cells (IECs) to mesenchymal like cells, upregulation of matrix metalloproteinases (MMPs) and overexpression of invasive molecules among the hypothetical pathways involved in the pathogenesis of fistulating CD.^7^^,^^8^ There is little evidence from genetic studies that clearly pinpoints a genetic source for this phenotype, except in the case of early onset monogenic inflammatory bowel disease (IBD).^9^^,^^10^ The contribution of somatic mutations to this aspect of CD is unknown. Finally, the role of the microbiota or environmental factors to fistulae remains unclear.

Here, we present a synthesis of the current knowledge on the pathogenesis of CD-associated fistulae while exploring how we might utilize advancing technologies to expand our understanding of the pathophysiological pathways determining fistulating CD.

## Histopathogenesis

**Figure 1:**
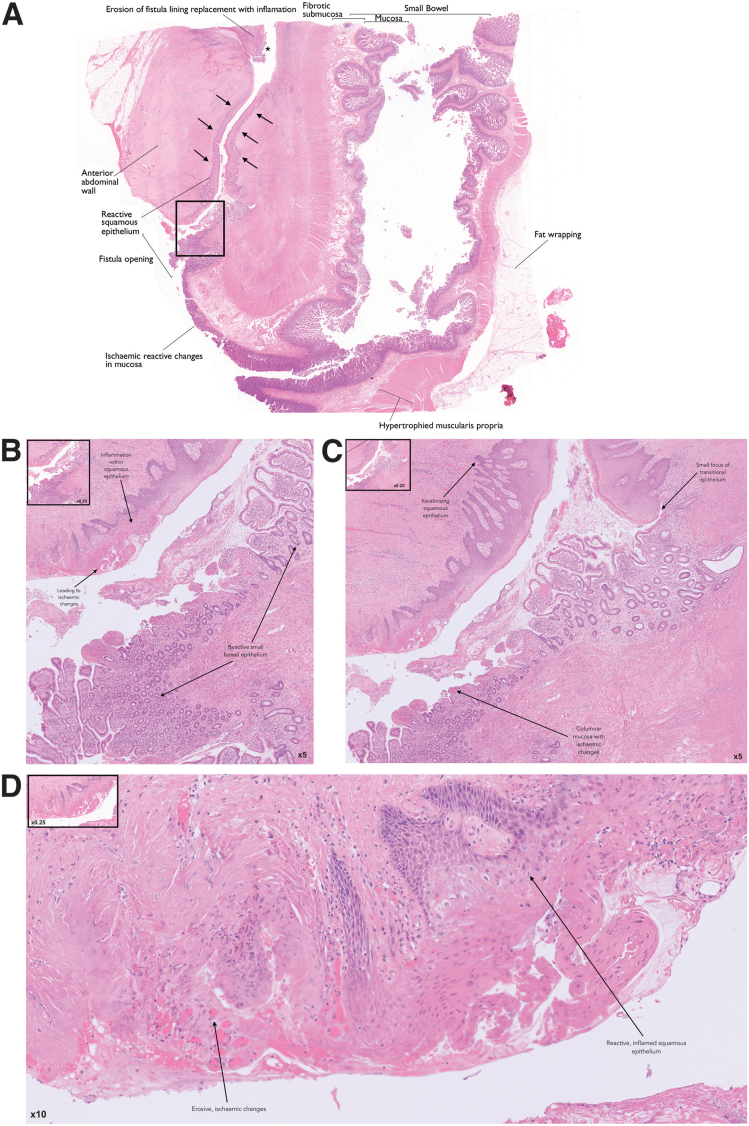
**Histopathological image of CD-associated enterocutaneous fistula.***A*, At low magnification, the central fissure of the fistula tract can be identified (*arrows*) between the anterior abdominal wall and ileum. On the ileal side of the fistula, the mucosa is destroyed with flattened columnar epithelium due to ischemic/reactive changes in the mucosa. On the cutaneous side of the fistula, features of reactive, narrow squamous epithelium is observed. As observed in the majority of fistulae, erosion of fistula lining is replaced with an inflammatory infiltrate (∗). Chronic fibrosis, which is a histopathological feature of CD-associated fistulae, is characterized here by hypertrophied muscularis propria and fibrotic submucosa within the ileum. Fat wrapping (or creeping fat), which is pathognomonic of ileal CD, is also observed in this specimen (×0.25 original magnification). *B*, On the ileal side of the fistula, reactive ileal epithelium may be observed. On the cutaneous side, inflammation may be observed within the squamous epithelium leading to ischaemic changes (×5 original magnification). *C*, A small focus of transitional epithelium is observed lining the fistula at ×5 magnification. In addition, keratinising squamous epithelium and ischaemic columnar mucosa is seen on the cutaneous side and ileal side of the fistula respectively (×5 original magnification). *D*, Further erosive ischaemic and inflamed changes seen at higher resolution (×10 original magnification) (hematoxylin-eosin).

Defining CD-associated fistulae histopathologically is vital to understanding its pathogenesis. A study examined several fistula specimens, both CD-associated and controls, histopathologically.^11^ All fistulae, independent of underlying cause, were characterized by a central fissure penetrating the lamina propria and muscularis mucosae through to underlying tissue layers. Universally, fistulae were surrounded by granulation tissue with histiocytes and a dense network of capillaries. The luminal contents may include debris, erythrocytes, or non-specific acute or chronic inflammatory cells.^11^ Granulomas may be present in CD-associated fistulae; however, they are not disease-specific: for instance, granulomas are absent in most CD-associated perianal fistulae.^12^ Chronic fibrosis may also be a histopathological feature of CD-associated fistulae (Figure 1).

Epithelium lining fistulae is typically flattened in the small or large intestine or consists of narrow squamous cells in perianal or cutaneous fistulae (Figure 1). Not all fistulae have an epithelial lining; however, two-thirds are non-epithelialized. Non-epithelialized CD-associated fistulae are instead lined by mesenchymal-like cells, termed ‘transitional cells,’ with retained gap junctions to each other. This thin layer of transitional cells (TCs) represents epithelial cells thought to have undergone transformation to mesenchymal-like cells. In some sections, a new basement membrane form with these TCs connected by fibronexus. In other sections, TCs may appear more disordered with no visible gap junctions and fragmented basement membranes. These features appear unique to CD-associated fistulae when compared with controls.^11^

The majority of fistula specimens demonstrate inflammation, with severe acute inflammation in at least 56%.^11^ Inflammatory cell populations have been studied immunohistochemically; in patients with CD-associated fistulae, the inner fistula wall is densely infiltrated with CD45RO^+^ T cells and a small band of CD68^+^ macrophages. The outer fistula wall is marked by CD20^+^ B cells. This is in contrast to non-CD-associated fistulae, whereby CD68^+^ macrophages are seen throughout the whole fistula wall, with CD45RO^+^ T cells occupying the outer two-thirds of the fistula wall. There are also considerably fewer B cells. The distribution of immune cells is independent of the fistulae location or the depth of penetration.^11^

Maggi and colleagues performed phenotypic and functional analysis of T-cells recovered from CD perianal fistulae tissue and circulating T-cells from peripheral blood^13^; CD4^+^CD161^+^ T-cells with Th17, Th1, Th17/1 phenotype significantly infiltrate fistula tissue compared with peripheral blood. Locally administered anti-TNF (tumor necrosis factor) (adalimumab) resulted in a resolving fistula clinically and a significant reduction in the frequency of CD161^+^ T-cells within fistula tissue.

A further study identified significant differences in T-cell subsets in fistulating CD when compared with healthy and non-fistulating CD; specifically, the authors detected an increase of CD3^+^CD8^-^ T-cells and a decrease in CD3^+^CD8^+^ T-cells in peripheral blood. Both T-cell subsets secreted high amounts of TNF-α and interleukin (IL)-13 in co-culture experiments, both of which are recognized as key cytokines in the pathogenesis of fistulating CD (Figure 1).^14^

## Immune and Cellular Biology

### The Epithelial-to-mesenchymal Transition Theory

Epithelial-to-mesenchymal transition (EMT) is a process by which an epithelial cell differentiates into a mesenchymal-like cell, acquiring its features and properties. Epithelial cells lose their defining characteristics, such as polarity and adhesiveness, and adopt a mesenchymal phenotype, such as reduced cell-to-cell adhesion and enhanced migratory potential.^15^^,^^16^

In health, EMT plays a critical role in embryogenesis and organ development.^17^^,^^18^ EMT is associated with the ability to migrate and penetrate into adjacent tissue layers. In disease, EMT may be seen contributing to cancer and fibrosis.^19^, ^20^, ^21^ EMT plays a pivotal role in tissue remodeling, occurring in response to tissue damage where there is a need to recruit mesenchymal cells from epithelial cells. Although the epithelial cells acquire the ability to migrate to sites of injury through EMT, dual effects of excess extracellular matrix (ECM) deposition secreted by mesenchymal like cells can result in tissue fibrosis.^22^ The shift from epithelial to mesenchymal cell is orchestrated by a wide range of cellular changes, such as expression of transcription factors, cytokines, and regulatory proteins.^9^

**Figure 2:**
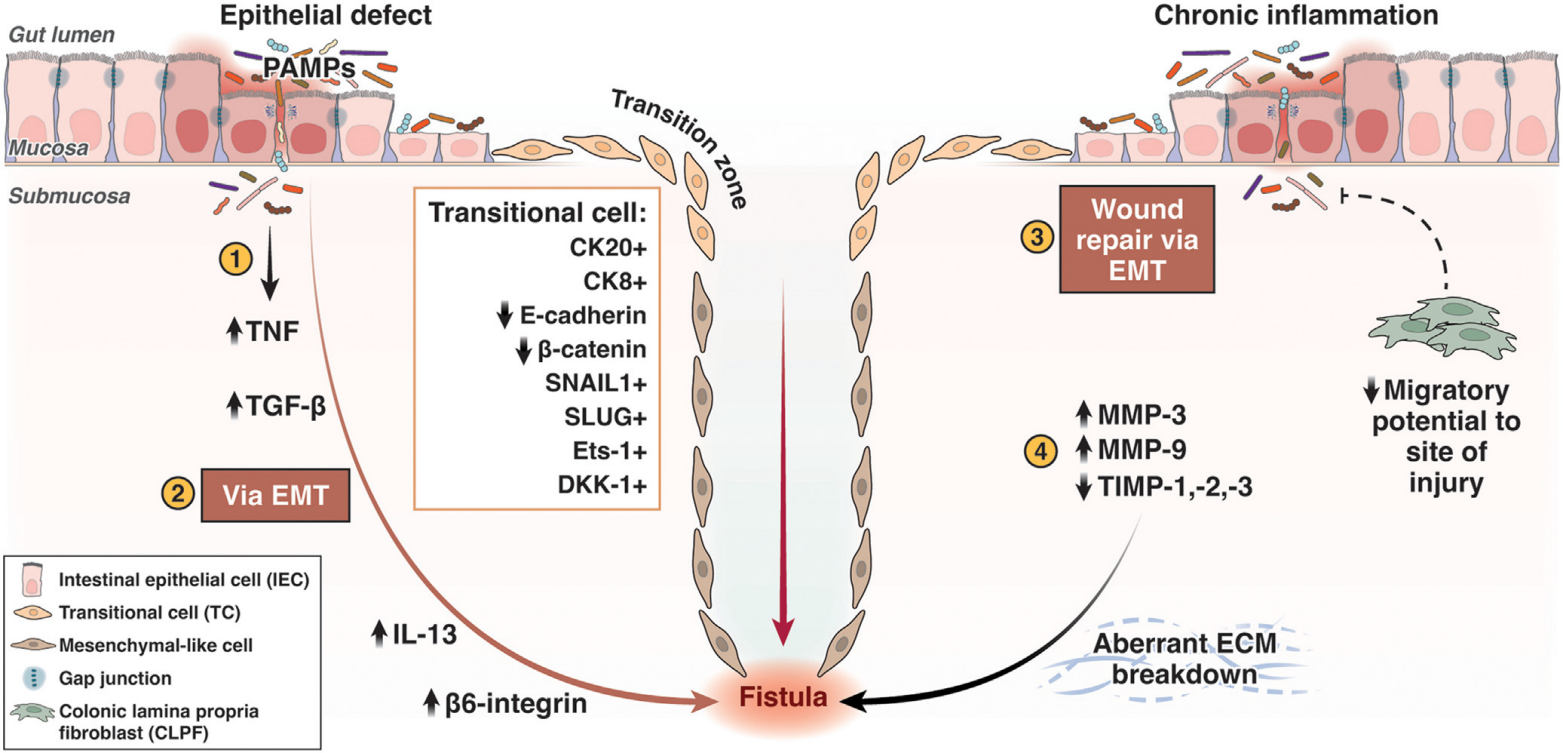
**Schematic of the pathogenesis of CD-associated fistula formation.** (1) IECs undergo EMT converting to TC in response to a defect in the epithelial barrier; resultant infiltration of pathogen associated molecular patterns enter the gut mucosa, which elicits an immune response. (2) An upregulation of TNF, a potent inducer of TGF-β, occurs which stimulates a cascade of pro-inflammatory cytokines (IL-13) and cell invasive molecules (β6-integrin) resulting in TCs adopting features of an invasive mesenchymal-like cell. TCs preserve their epithelial origins (CK20/8+); however, they down-regulate expression of epithelial cell adhesion molecules (E-cadherin, β-catenin) and highly express EMT-inducing transcription factors (SNAIL1, SLUG, Ets-1) and cell migratory molecule, DKK-1. In addition, TCs may appear more disordered with a loss of gap junctions and fragmented basement membranes. (3) Chronic inflammation results in reduced epithelial repair and reduced migratory capabilities of CLPFs, which contribute to poor wound healing in fistulating CD; IECs compensate by undergoing EMT in an attempt to restore the epithelial barrier. (4) MMPs (MMP-3, MMP-9) are highly expressed and unopposed (reduced TIMP-1, -2, -3) in fistulating CD tissue, resulting in aberrant breakdown of the extracellular matrix and tissue remodeling. These pathways consequently contribute to fistula formation. (Figure created with BioRender.com and adapted from Siegmund et al.^12^ CK, Cytokeratins; PAMP, pathogen-associated molecular pattern.

Much data provides supporting evidence for the theory that EMT drives CD-associated fistula formation.^11^^,^^23^^,^^24^ Chronic inflammation results in both reduced epithelial repair and reduced migratory capabilities of colonic lamina propria fibroblasts (CLPFs), which contribute to poor wound healing in fistulating CD.^25^^,^^26^ Within a fistula, IECs compensate for defective CLPFs by converting to TCs in an attempt to restore the intestinal epithelial barrier.^11^^,^^27^ TCs form a thin monolayer, lining the fistula. The region where IECs transform into TCs is referred to as the ‘transitional zone.’ TCs appear unique to CD-associated fistulae. Present in ‘non-epithelialized’ areas of fistula, TCs express epithelial markers cytokeratin 8 and 20, reflecting their epithelial origins. Expression of epithelial adhesion markers, E-cadherin, and β-catenin is downregulated, however.^23^ Both proteins facilitate cell-to-cell adherence; therefore, their downregulation allows the cell to adopt migratory properties – a defining role of EMT (Figure 2).

Previous studies defining CD-associated fistulae immunohistochemically, have demonstrated the presence of potent inducers and markers of EMT.^23^^,^^28^ β6-integrin is overexpressed in the transitional zone of TCs in CD-associated fistulae^23^; this overexpression of β6-integrin correlates with increased cell invasiveness.

Severe intestinal inflammation results in the secretion of cytokines TNF-α, IL-13, and transforming growth factor-beta (TGF-β ). TGF-β is the most potent inducer of EMT. It results in the induction of transcription factors in IEC associated with EMT such as E-twenty-six (Ets)-1, SNAIL1, and SLUG (SNAIL2). The SNAIL family transcription factors strongly repress E-cadherin. SNAIL1 has been shown to be highly expressed in the nuclei of TCs lining CD-associated fistulae; SLUG (SNAIL2) in contrast is mainly limited to cells around the fistula tract.^29^ In addition, Ets-1 is highly expressed in CD-associated fistulae.^24^^,^^30^ Ets-1 mediates the activation of β 6-integrin and therefore enhances cell invasion during EMT. Upregulation of TGFβ-1 and TGFβ-2 expression is also seen in TCs lining CD-associated fistula tracts when compared with normal IECs.^23^ Its upregulation is also linked to β 6-integrin expression.^23^^,^^31^

In summary, strong features to suggest EMT in CD-associated fistulae include reduced E-cadherin and β-catenin expression, upregulation of TGF-β, induction of EMT transcription factors (SNAIL1, SLUG, and Ets-1), and β6-integrin overexpression in TC – all resulting in enhanced migratory potential and increased cell invasiveness. These features appear to be consistent, irrespective of fistula location^16^ (Figure 2).

Dickkopf-related protein 1 (DKK-1) is an important factor in the regulation of cell migration through its ability to block IEC migration. Expression of DKK-1 is strongly upregulated in the TCs lining CD-associated fistula tracts, with weak expression in healthy controls.^32^^,^^33^ New findings propose its function as a Wnt inhibitor may regulate TGF-β-stimulated IL-13 secretion and therefore EMT in IECs.^33^

More recently, Ortiz-Masiá and colleagues demonstrated that the levels of metabolite succinate and expression of its receptor SUCNR1 were significantly increased in fistulating CD tissue. SUCNR1 increases expression of Wnt ligands and activates Wnt signaling pathways, which in turn induces EMT in IECs.^28^ Previous work from the same authors shows that increased EMT induction in fistulating CD tissue is driven by an increased interaction between Wnt ligand, WNT2b, and receptor FDZ4 when compared with controls.^32^

## Cytokine Profile

Published data has sought to define the cytokine profile of CD-associated fistula tracts and therefore glean its immunopathogenesis. TNF induces EMT in IECs and can induce expression of TGF.^30^^,^^34^^,^^35^ TNF and its receptor, TNF receptor 1 (TNF-R1), are strongly expressed in TCs lining fistulas along with IECs of adjacent crypts in patients with CD.^29^ In addition, in both IECs and CLPFs, TNF induces β6-integrin and Ets-1 transcription factor, both key mediators of EMT.^30^ Furthermore, serum TNF-α levels significantly correlate with the presence of active CD perianal fistulae.^36^

Similar to TNF, IL-13 and its receptor, IL-13R1, are heavily expressed in TCs lining fistula tracts and adjacent crypts. This appears unique to CD-associated fistulae, as IL-13 expression is notably absent in healthy intestine, ulcerative colitis, and non-fistulating CD, regardless of inflammation. IL-13 upregulates TNF-α, IL-12, and IL-6 in fistulating tissue.^24^ Functionally, IL-13 promotes genes involved with cell invasion (β6-integrin) and EMT (SLUG) in IEC and in in vitro models of EMT.

A study examining cytokine concentrations in fistula tracts of idiopathic vs perianal CD fistulae demonstrated significantly higher IL-12 concentrations and a lower IL-1RA/IL-1β ratio in the CD group.^37^

TGF-β, the key mediator of EMT, also localizes to the TCs lining fistula tracts and induces SNAIL1 and IL-13 in in vitro models. Primary human CLPFs derived from patients with fistulating CD demonstrated altered function when treated with TGF-β. TGF-β and IL-13 are thought to display synergism in the pathogenesis of fistulae.^24^

The abundance of these cytokines in the lining of fistula tracts, adjacent tissue, and peripheral blood implies their involvement in fistulating CD pathogenesis. Furthermore, the significance of the role of cytokines is further substantiated by the clinical efficacy of anti-cytokine biological agents such as infliximab (anti-TNF-α) in the treatment of fistulating CD.^38^

### Matrix Metalloproteinases and Tissue Inhibitors of MMP

MMPs have tissue degrading and remodeling properties. Aberrant ECM breakdown secondary to MMP activity can lead to cancer or IBD. Increased MMP activity is associated with immune-mediated tissue injury and is found in CD.^39^ Murine models of dextran sulfate sodium-colitis demonstrate the significance of MMPs, with selected deletion of MMP-9 conferring a protective effect.^40^, ^41^, ^42^, ^43^ Tissue inhibitors of MMP (TIMPs) are the natural inhibitors of MMPs, secreted by MMP-producing cells.^44^ Kirkegaard and colleagues identified strong expression of MMP-3s in CD-associated fistula tissue when compared with controls, irrespective of inflammatory state. MMP-3s were largely localized to mononuclear cells and fibroblasts, with MMP-9 predominantly in granulocytes and only in fistulae with active inflammation.^45^ MMP-3 and MMP-9 have also been identified in idiopathic fistulae. MMP-13 protein expression is also detectable in CD-associated fistulae but almost absent in non-fistula CD tissue. Protein levels of inhibitory molecules TIMP-1, TIMP-2, and TIMP-3 are correspondingly low in CD-associated fistulae tissue.^45^ This provides evidence for the basis of MMPs as mediators in the pathogenesis of CD-fistulae through aberrant ECM degradation (Figure 2).

### Genetic Contribution

The interactions between genetics and environment are well described in CD. Extensive genome-wide association studies have identified approximately 240 genes associated with pathogenesis or risk of disease.^46^, ^47^, ^48^ Genetic contributions per fistulating phenotype have also been described.^49^, ^50^, ^51^ Nucleotide oligomerization domain 2 (*NOD2*) remains the strongest genetic predictor of CD susceptibility and phenotype including fistulating disease.^51^, ^52^, ^53^ Henckaerts at al demonstrated the following as independently associated with non-perianal CD fistulating phenotype; the presence of a T-allele at rs12704036 (odds ratio [OR], 1.74), followed by the presence of any *NOD2* variant (OR, 1.47), and *IRGM* rs4958847 (OR, 9.22).^49^

The presence of a C allele at *CDKALI* rs6908425 and the absence of any *NOD2* variant was associated with perianal fistulating phenotypes (*P* = .008 and *P* = .002, respectively).^49^ Conversely, in latter work, *NOD2* mutation, rs72796353 was found to be significantly associated with perianal fistula development (OR, 5.27; *P* = 2.78 × 10^-7^) when compared with *NOD2* wild-type carriers.^54^

In addition to *NOD2* polymorphisms, the risk of developing internal penetrating CD is significantly associated with the carriage of a variant allele of *PRDM1* rs7746082, LOC441108, and *IL23R.* Specifically, the carriage of *ATG16L1* and *PRDM1* are independently associated with an earlier onset of internal penetrating disease in contrast to *IL23R*, which is associated with a later onset.^51^ OCTN variants have also been demonstrated in association with penetrating disease behavior in both Korean (OR, 4.23)^55^ and Belgian (OR, 1.47)^56^ populations. The gene *PUS10* confers a protective effect against perianal fistulizing disease.^51^

More recently, epigenetic analysis defined an intestinal mucosa-derived DNA methylation signature in fistulating CD mucosal lesions; differential DNA methylation sites were enriched in the upregulation of apoptotic processes and IL-8 production when compared with normal intestinal mucosal tissue.^57^

It is noteworthy that genetic contributions to the CD fistulating phenotype differ in perianal and internal fistulae. However, universally, the risk alleles associated with fistulating CD encode for proteins involved with regulation of ileal microbiota, adaptive immunity, or maintaining the integrity of the intestinal epithelial barrier.

### Microbial Contribution

The host-microbe interaction in CD is well-studied^48^^,^^58^; less well-characterized is the role of the intestinal microbiome in CD-associated fistula development and persistence. There is a rationale to suggest a bacterial contribution to the etiology of fistulae, given the efficacious role of antibiotics in its management, namely in perianal fistulae. A limited number of studies have explored the microbial composition of CD-associated fistula tracts to characterize potential causative organisms. In a cohort of 13 patients with CD-perianal fistulas, West and colleagues demonstrated that perianal fistulae are predominantly colonized with gram-positive microorganisms.^59^ Another study showed that gram-positive bacterium *Corynebacterium* and gram-negative *Achromobacter* were significantly more abundant in perianal fistula tract samples relative to matched stool samples via 16S rRNA gene profiling.^60^ In contrast, a study examining idiopathic and Crohn’s anal fistula tracts did not isolate any mucosa-associated bacteria despite the presence of mucosal inflammation. Although the authors acknowledged potential for sampling error, they also suggest that established fistula tracts devoid of bacterial colonisation may imply that bacteria are unimportant for fistula persistence.^61^

Bacterial cell wall muramyl dipeptide, for which *NOD2* is a receptor for, induces expression of molecules relevant to EMT (TNF-α, TGF-β, SNAIL1, IL-13, and Ets-1) within IECs and lamina propria fibroblasts from fistulae.^30^ Furthermore, it has been reported that certain enteric pathogens, such as *Citrobacter rodentium* and *E. coli*, can trigger the onset of EMT through activated signaling pathways, implying that luminal bacteria may play a role in fistula development through the theorized EMT pathway.^62^^,^^63^

Emerging data is now defining the role of the mycobiome in CD; Jain et al demonstrate that fungus Debaryomyces hansenii is enriched in inflamed intestinal CD tissue and can cause dysregulated mucosal healing.^64^ Such alterations in tissue repair capacity may contribute to the pathogenesis of CD-associated fistula. The natural competition between fungi and bacteria within the gut is disturbed with the use of antibiotics. It is feasible that, with the elimination of certain bacteria, impaired mucosal healing may occur due to the unopposed action of and relative enrichment of fungi within the gut and may give explanation to the persistence of perianal fistulae, in some cases, despite antibiotics.

With advancing technologies, future work needs to both refine and broaden our understanding of the potential role of the microbiome, including the mycobiome, in CD-associated fistulae.

### Future Avenues

Historically, perianal CD-associated fistulae have been better phenotyped principally due to easier access to tissue. Direction of future work must first begin with accessing lesional tissue from CD-associated intestinal fistulae. Using novel methods, researchers must first phenotype human lesional tissue and define the microenvironment of CD-associated fistulae. The understanding of which will inform the conditions or pathways in which in vivo models might be developed and explored. Secondly, leveraging existing knowledge of the phenotypic and molecular characteristics of idiopathic fistulae^45^^,^^65^^,^^66^ will be a first flourish in understanding the distinct and shared pathways of CD-associated fistula pathogenesis.

### In Vitro Models

To date, the use of in vitro models (eg, intestinal epithelial cell lines) and descriptive histopathological and immunohistochemical data has been the main source of knowledge of fistulating CD pathology.^14^^,^^23^^,^^32^ Meier and colleagues successfully developed a 3-dimensional matrix model to study the behavior of CD-CLPFs isolated from CD-associated fistulae and strictures. Patient-derived CLPFs were cultured and seeded into a 3-dimensional matrix; the CLPF layer underwent laser wounding with subsequent study of cell migration assays. Fistula-associated CLPFs were found to have significantly reduced migratory potential.^27^

Remarkable advances in the development of intestinal epithelial organoids (IEOs) now offer a more accurate representation of intestinal epithelial structure and function in health and disease.^67^^,^^68^ Organoid-based models have also provided further evidence for EMT in intestinal diseases such as CD.^69^, ^70^, ^71^, ^72^, ^73^ Pivotal work from Hahn et al demonstrated the ability of TNF-α and TGF-β to induce EMT in IEOs; mesenchymal phenotypic changes were observed in the TGF-β1-stimulated IEOs. proposing a novel model for studying EMT in intestinal fibrosis.^69^

Moreover, sequencing technology advances permit identification of somatic mutational burden within epithelia of patient-derived organoid cultures.^74^ Future work should focus on developing a reproducible intestinal fistula-derived organoid model including both epithelial and stromal compartments. By manipulating the inflammatory microenvironment in such organoids, future work will clarify the possible contribution of EMT in CD-associated fistulae formation and explore whether said process may be stopped or indeed reversed.

Finally, recent advances in tissue engineering and organoid methodologies might serve as an exciting prospect to model CD-associated fistulae in vitro. Meran et al successfully generated decellularized intestinal tissue scaffolds with resected human intestines seeded with patient-derived intestinal organoids.^75^ Leveraging such methods could be the key next step in model development.

### In Vivo Models

However, a major limitation in the field of fistulating CD research is the lack of a suitably sufficient and reproducible animal model. Only a few animal models have shown promise in their ability to develop intestinal fistulae. Firstly, Cominelli’s group established a sub strain of the SAMP1/Yit mouse associated with spontaneous perianal fistula formation.^76^ The SAMP1/YitFc mouse, generated by sibling mating over 20 generations, exhibited fistulating perianal disease in 4.8% of the colony. The disease was characterized by mucosal ulceration of the anal canal, fissures, perirectal abscesses, and anocutaneous fistulae. Multiple fistulae were not observed, however. Although this model is arguably the closest animal model of CD with its co-existing terminal ileitis, the low incidence of perianal fistula limits its widespread use as a fistulating model.

Enterocutaneous fistula formation in a human gut xenograft is another novel model whereby Bruckner and colleagues^77^ transplanted human fetal gut segments subcutaneously into mature SCID (C.B-17/IcrHsd-Prkdcscid) mice. Approximately 17% developed spontaneous enterocutaneous fistulae, notably in those with a lack of IL-10 response. Histopathological findings showed remarkable similarities with CD-associated fistulae, including evidence for EMT immunohistochemically.^78^ Although promising, this model requires further validation and replication. Several mouse models of non-intestinal fistula exist including trachea-esophageal^79^ and vascular fistulae,^80^ which may also serve to inform development of future CD models.

## Conclusions

Fistulating CD is a complex clinical entity with significant morbidity and mortality to the patient and economic burden to health care. Development of targeting treatment for fistulae will be dependent on a more in-depth understanding of pathogenesis. The most accredited theory for its pathogenesis remains EMT, with several inducers of EMT identified in CD-associated fistula tissue and validated with in vitro models. To date, data on EMT has largely been captured by reverse transcription polymerase chain reaction, co-culturing, and immunohistochemistry techniques. Multimodal single-cell analysis will enable definition of cell circuits driving this phenomenon. Understanding the contribution of the tissue niche around fistulating tissue and epithelial-stromal crosstalk unique to fistulating CD will be key to advancing the field.

Although aberrant epithelial-stromal crosstalk is important, the wider cellular milieu also plays a role. Recent data on the significance of the mesentery in the pathogenesis CD is of interest^81^; the mesentery, including lymphatics, adipose tissue, and nerves, host metabolic and immune properties,^82^ and may indeed have a role in fistulating CD. The exact mechanisms of which are unknown and warrant further study.

The differential risk of fistulating CD by ethnicity is considered with interest; the contribution of environment and genetics by ethnicity is unclear. Although a single genetic source does not pinpoint the fistulating phenotype, it is noteworthy that much of the literature on genetic contributions in IBD is derived from Caucasian populations. Similarly, the role of environmental factors in fistulae formation is underdeveloped.

In summary, our current understanding suggests that the pathogenesis of fistulating CD is multifactorial – a combination of EMT and overexpression of MMPs in response to an epithelial defect mediated by the upregulation of pro-inflammatory cytokines, genetic susceptibility, epigenetic changes, and possible influence of the intestinal microbiota. The development of robust in vivo model systems of fistulating CD is required to verify and broaden our current understanding while exploring novel causal pathways to provide further inroads to the pathogenesis of fistulating CD.
